# *In Silico* Approach for the Definition of radiomiRNomic Signatures for Breast Cancer Differential Diagnosis

**DOI:** 10.3390/ijms20235825

**Published:** 2019-11-20

**Authors:** Francesca Gallivanone, Claudia Cava, Fabio Corsi, Gloria Bertoli, Isabella Castiglioni

**Affiliations:** 1 Institute of Molecular Bioimaging and Physiology, National Research Council (IBFM-CNR), Via F. Cervi 93, 20090 Segrate-Milan, Milan, Italy; francesca.gallivanone@ibfm.cnr.it (F.G.); claudia.cava@ibfm.cnr.it (C.C.); isabella.castiglioni@ibfm.cnr.it (I.C.); 2 Laboratory of Nanomedicine and Molecular Imaging, Istituti Clinici Scientifici Maugeri IRCCS, via Maugeri 4, 27100 Pavia, Italy; fabio.corsi@icsmaugeri.it; 3 Department of Biomedical and Clinical Sciences “L. Sacco”, Università degli studi di Milano, via G. B. Grassi 74, 20157 Milano, Italy; 4 Breast Unit, Surgery Department, Istituti Clinici Scientifici Maugeri IRCCS, via Maugeri 4, 27100 Pavia, Italy; 5 Department of Physics “Giuseppe Occhialini”, University of Milan-Bicocca, 20126 Milan, Italy

**Keywords:** radiogenomics, RadiomiRNomics, breast cancer, magnetic resonance imaging, MRI, microRNAs/miRNAs, pathways, network

## Abstract

Personalized medicine relies on the integration and consideration of specific characteristics of the patient, such as tumor phenotypic and genotypic profiling. Background: Radiogenomics aim to integrate phenotypes from tumor imaging data with genomic data to discover genetic mechanisms underlying tumor development and phenotype. Methods: We describe a computational approach that correlates phenotype from magnetic resonance imaging (MRI) of breast cancer (BC) lesions with microRNAs (miRNAs), mRNAs, and regulatory networks, developing a radiomiRNomic map. We validated our approach to the relationships between MRI and miRNA expression data derived from BC patients. We obtained 16 radiomic features quantifying the tumor phenotype. We integrated the features with miRNAs regulating a network of pathways specific for a distinct BC subtype. Results: We found six miRNAs correlated with imaging features in Luminal A (*miR-1537*, *-205*, *-335*, *-337*, *-452*, and *-99a*), seven miRNAs (*miR-142*, *-155*, *-190*, *-190b*, *-1910*, *-3617*, and *-429*) in HER2+, and two miRNAs (*miR-135b* and *-365-2*) in Basal subtype. We demonstrate that the combination of correlated miRNAs and imaging features have better classification power of Luminal A versus the different BC subtypes than using miRNAs or imaging alone. Conclusion: Our computational approach could be used to identify new radiomiRNomic profiles of multi-omics biomarkers for BC differential diagnosis and prognosis.

## 1. Introduction

The advent of new gene expression profiling using microarray and next generation sequencing technologies has allowed scientists to investigate the molecular complexity of breast cancer (BC). These studies identified gene signatures, such as the 70-gene MammaPrint microarray assay (Agendia, Amsterdam, the Netherlands) [1,2], The 21-gene Oncotype DX assay (Genomic Health, Redwood City, Ca, USA) [3,4,5], and the 50-gene PAM50 assay (Prosigna, Nanostring Technologies, Seattle, WA, USA) [6,7], which are useful in the management of BC by predicting prognosis and/or efficacy of treatment. However, molecular characterization using genomic and proteomic technologies requires invasive sampling of tissue portions. The analysis of single portions of tissue, not representative of the entire tumor, limits the impact of characterization of oncological malignancies, which are both spatially and temporally heterogeneous.

MicroRNAs (miRNAs) emerged as possible disease biomarkers, also in the form of circulating molecules, being extremely stable in serum, plasma, and urine, and easy to access by non-invasive tests [8]. MiRNAs, which are short (18–22 nucleotides) non-coding RNA sequences, are novel biomarkers of breast tumorigenesis, with tissue-specific expression, stability in biofluids, and the ability to regulate hundreds of genes and biological pathways [8]. They are appealing targets for screening, diagnosis, prognosis, discovery, and the choice of correct treatment for patients [8,9].

Molecular imaging technologies, such as magnetic resonance imaging (MRI), play a central role in the management of oncological pathologies, due to their ability to in vivo and non-invasively characterize the presence of disease and visualize the entire tumor volume at different time points, enabling the monitoring of tumor growth and response to therapy [10]. With respect to genomic data, imaging can sample the entire tumor volume to allow assessing the tumor phenotype in vivo [11]. In standard clinical practice, dynamic contrast-enhanced MRI (DCE-MRI) is routinely used for BC diagnosis, screening, and follow-up monitoring [12,13,14]. Whereas, DCE-MRI is an inherently quantitative technique, radiologists still use a semantic lexicon to describe imaging phenotype. However, the availability of advanced image processing methods stimulated the development of quantitative approaches. Within this context, radiomics emerged as a methodology to convert images into quantitative descriptors of tumor phenotype and to mine these features to obtain an imaging signature for disease characterization or response to therapy prediction. In radiomics, the imaging phenotype provides information about underling histopathology and genetics, fully depicting the tumor and the tumor microenvironment [15].

Imaging-derived indices may help better characterize the whole tumor than biopsy alone. However, these indices do not provide information about the altered biological functions underlying the observed phenotype. A new area of research has emerged, called radiogenomics [16]. Radiogenomics refers to the integration of image-extracted phenotypes and genomic data of the same tumor, which has the potential to improve both diagnosis and therapeutic strategies for evaluating the individualized disease signatures with higher accuracy.

Since the advent of imaging in diagnostic workup of oncological pathologies, the relationship between the imaging pattern of the disease and the underlying biology were explored. The first attempt was made in 2007 by Segal et al. [17] who, using an advanced and intensive computational approach, described the tentative correlations between features extracted by a semantic lexicon, global gene expression patterns, and prognosis in liver cancer. Since then, several other attempts have been made, including correlating imaging features, gene expression modules, and prognosis in brain tumors [18]; positron emission tomography-computer tomography (PET-CT) imaging features; and gene expression profiles and survival in non-small cell lung cancer [19]. The possibility of using advanced computational approaches to image processing have motivated studies [20] and the term radiomics emerged, as a high-throughput methodology to extract accurate quantitative descriptors of imaging tumor phenotype, capturing biological tumor heterogeneity. With radiomics, molecular imaging techniques can be used to assess intra-tumoral heterogeneity, as the translation of tumor genomic heterogeneity, allowing the identification of patients that would ultimately exhibit worse prognosis. This has been confirmed by other works, such as Segal et al. [17] who showed that the combination of only 28 imaging traits from CT was sufficient to reconstruct the variation of 116 gene expression modules in hepatocellular carcinoma. Diehn et al. [18] showed that in glioblastoma multiform (GBM), proliferation and hypoxia gene expression patterns can be predicted by mass effect and tumor contrast enhancement as evaluated by MRI. They also showed that a specific imaging pattern could predict overexpression of epidermal growth factor receptor (EGFR) and that the infiltrative pattern of T2 hyperintense signal on MR images was highly predictive of outcome. The authors concluded that MR imaging could provide an in vivo portrait of genome-wide gene expression in GBM.

In the subsequent years, focusing mainly on imaging, several groups explored the signatures of intratumor heterogeneity captured by quantitative molecular imaging techniques to define patient prognosis and response to therapy in different cancers [21,22]. Despite this, the image phenotype of intratumor heterogeneity was associated to omics and clinical data in few published works [11,23], often including a limited number of patients [15]. Considering BC, Antunovic et al. [24] demonstrated that PET/CT features are correlated to molecular BC subtypes obtained by immunohistochemistry, suggesting that radiomics could be successfully applied to the identification of molecular subtypes. In an initial study, Leithner et al. [25] evaluated DCE-MRI for the assessment of BC receptor status and molecular subtypes, obtaining a high diagnostic accuracy. The possibility of applying machine learning techniques to radiomics analysis has improved the diagnostic results in several cancer types [26,27,28], even though different methodological issues have to be addressed and the number of involved patients often limits the possibility of developing and assessing predictive models to be tested with artificial intelligence methods.

The aim of our study was to classify the four BC subtypes (Luminal A, Luminal B, HER2+, and Basal) using epigenomic miRNA (miRNomic) markers and radiomic imaging features. In this radiomiRNomic study, we explored the integration of gene and miRNA expression profiles with radiomic features of BC subtypes derived from The Cancer Genome Atlas (TCGA) [29] and The Cancer Imaging Archive (TCIA) [30]. In particular, we used the TCGA/TCIA-BRCA_1 dataset, which contains both miRNomic profile and radiomics features, and the TCGA/TCIA-BRCA_2 and GSE81000 datasets, containing only radiomics data and only miRNA expression data, respectively. The TCGA/TCIA-BRCA_1 data set was used to identify the relationships between miRNAs and radiological phenotype; the TCGA/TCIA-BRCA_2 dataset was used for the validation of the obtained radiomic signature. For miRNA signature validation, we used an independent dataset, GSE81000. To the best of our knowledge, we are the first to investigate the relationships between the miRNAs-regulating pathway network and radiomics features in BC subtypes using a radiomiRNomic approach, assuming that radiomics of cancer lesions can express genetic and epigenetic features in its in vivo microenvironment. The purpose of our study was to provide a proof-of-concept of combining radiomics and miRNomic features to improve the classification results of BC subtypes. We identified a network of pathways containing differentially expressed genes (DEGs) specific for each BC subtype (Luminal A, Luminal B, HER2+, and basal) with respect to normal tissue with a system biology approach. Then, we found miRNAs with a statistically significant role in regulating the genes of the pathways’ networks that are best able to classify each of the four BC subtypes. In parallel, we extracted quantitative MRI features from each of the four BC subtypes and analyzed the association between miRNAs and radiomic features of the same patients. A machine learning approach was used to verify whether the combination of miRNomics and radiomics better predicts the BC subtype than using either of the two alone.

## 2. Results

### 2.1. Association Between miRNAs and Imaging Features

**Figure 1:**
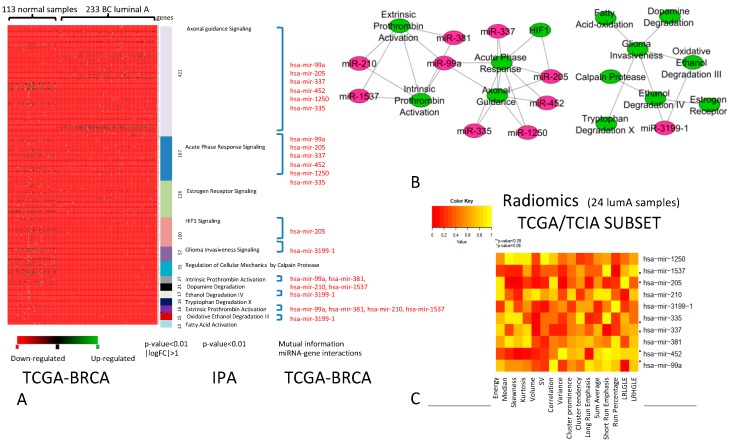
Breast cancer (BC) Luminal A. (**A**) The pathways able to classify Luminal A vs. normal samples (*p*-value < 0.01, |logFoldChange (FC)| > 1). For each pathway (in different colours), miRNAs (indicated in red) able to regulate differentially expressed genes (DEGs) are indicated. (**B**) Relationships between pathways and miRNAs (green nodes, pathways; purple nodes, miRNAs). (**C**) Heatmap of the correlation between imaging features and miRNAs. The color intensity in the figure shows the corresponding p-value; yellow cells indicate greater statistical significance.

The comparison between DEGs of Luminal A vs. normal samples revealed a net of differentially expressed pathways (Figure 1A) that consists of 13 pathways that were best able to classify luminal A BC vs. normal samples. The DEGs within these 13 pathways are potential targets of 10 miRNAs. Figure 1B shows the relationships between pathways and miRNAs, where the green nodes represent the pathways and purple nodes indicate the miRNAs. Edges in the network represent the cross-talk among pathways or the interaction between the miRNA and pathway. The analysis of correlation between imaging features and miRNAs regulating pathway cross-talk (miRNA-R), described by the heatmap in Figure 1C, revealed that 6 out of 10 miRNAs (*miR-1537*, *miR-205*, *miR-335*, *miR-337*, *miR-452*, and *miR-99a*) in Luminal A were significantly associated with imaging features (IFs). In particular, *miR-1537* was correlated with run percentage (RP) (*p*-value < 0.05) from the gray-level run length matrix (GLRLM), *miR-205* was correlated with median and skewness of the intensity-based histogram and variance (*p*-value < 0.05) from GLCM, and *miR-335* and *miR-337* were correlated with the volume, showing significant enhancement (*p*-value < 0.05). *miR-337* was also correlated with short run emphasis (SRE) from GLRLM (*p*-value < 0.05), *miR-452* was correlated with skewness from intensity histogram (*p*-value = 0.06), and *miR-99a* with long run low gray-level emphasis (LRLGLE) from GLRLM (*p*-value < 0.05).

**Figure 2:**
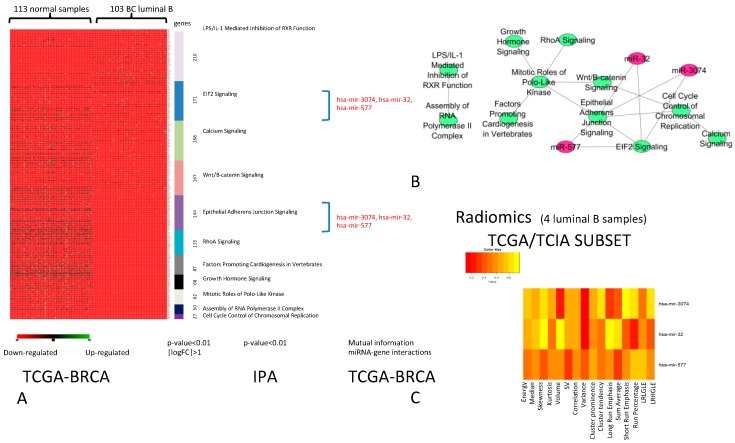
BC Luminal B. (**A**) Pathways able to classify Luminal B vs. normal samples (*p*-value < 0.01, |logFC| > 1). For each pathway (in different colours), miRNAs (in red) able to regulate DEGs are indicated. (**B**) Relationships between pathways and miRNAs (green nodes are the pathways and purple nodes are the miRNAs). (**C**) Heatmap of the correlation between imaging features and miRNAs. The color intensity in the figure shows the corresponding *p*-value; yellow cells indicate greater statistical significance.

The comparison between the DEGs of Luminal B and normal samples revealed a group of differentially expressed pathways (Figure 2A) that contains of 11 pathways that best classify of Luminal B BC vs. normal samples. We found three miRNAs (*miR-32*, *miR-577*, and *miR-3074*) regulating these pathways (Figure 2B). The analysis of correlation between IFs and miRNA-R, described by the heatmap (Figure 2C), revealed no significant correlation for Luminal B BC. For this subtype, further investigations are required due to the limited number of Luminal B samples.

**Figure 3:**
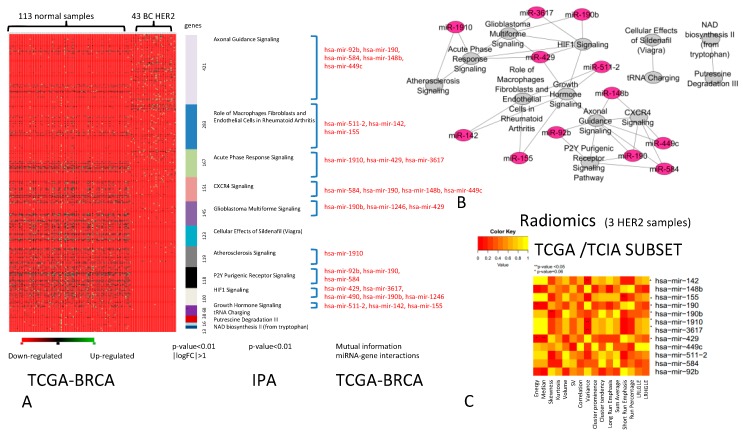
BC HER2+. (**A**) The pathways able to classify HER2+ BC vs. normal samples (*p*-value < 0.01, |logFC| > 1). For each pathway (indicated in different colours), miRNAs (in red) able to regulate DEGs are indicated. (**B**) Relationships between pathways and miRNAs (the green nodes are the pathways and purple nodes the miRNAs). (**C**) Heatmap of the correlation between imaging features and miRNAs. The color intensity in the figure shows the corresponding *p*-value; yellow cells indicate greater statistical significance.

The comparison between DEGs of HER2+ BC vs. normal samples revealed a group of differentially expressed pathways (Figure 3A) that consists of 13 pathways that were best able to classify HER2+ BC vs. normal samples. We found 12 miRNAs regulating these pathways, creating the network shown in Figure 3B. The analysis of correlation between IFs and miRNA-R, described by the heatmap (Figure 3C), revealed that for HER2+ BC, 7 out of 12 miRNAs (*miR-142*, *miR-155*, *miR-190*, *miR-190b*, *miR-1910*, *miR-3617*, and *miR-429*) had significant associations with IFs. In particular, *miR-142* was correlated with variance (VAR) (*p*-value < 0.05), *miR-155* was correlated with SRE from GLRLM (*p*-value < 0.05), *miR-190* was correlated with energy from the intensity histogram (*p*-value < 0.05), and *miR-190b* was correlated with skewness from the intensity histogram (*p*-value < 0.05). *miR-1910* and *miR-3617* were correlated with VAR from GLCM (*p*-value = 0.06 and < 0.05, respectively), whereas, *miR-429* was associated with the energy (*p*-value = 0.06) and median of the intensity histogram (*p*-value < 0.05). For this subtype, further investigations are required due to the limited number of HER2+ samples.

**Figure 4:**
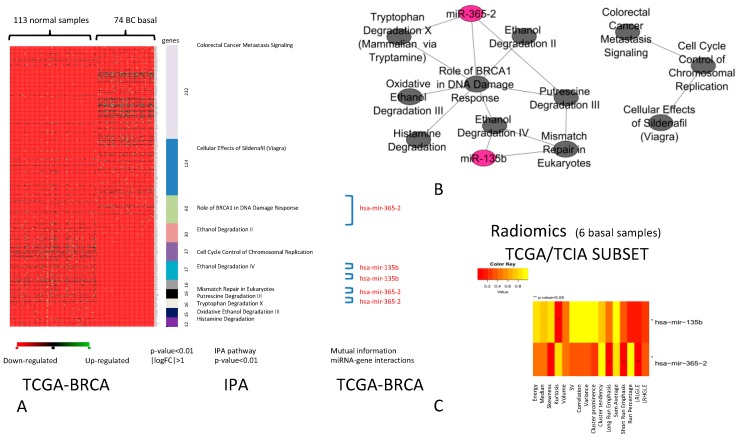
BC Basal. (**A**) The pathways able to classify basal BC vs. normal samples (*p*-value < 0.01, |logFC| > 1). For each pathway (in different colours), miRNAs (in red) able to regulate DEGs are indicated. (**B**) Relationships between pathways and miRNAs (the green nodes are the pathways and purple nodes are the miRNAs). (**C**) Heatmap of the correlation between imaging features and miRNAs. The color intensity in the figure shows the corresponding p-value; yellow cells indicate greater statistical significance.

The comparison between DEGs of basal BC vs. normal samples revealed a group of differentially expressed pathways (Figure 4A) that consists of 11 pathways that obtained the best performance in the classification of basal BC vs. normal samples. We found two miRNAs (*miR-135b* and *miR-365-2*) regulating these pathways, creating the network shown in Figure 4B. The analysis of correlation between IFs and miRNA-R, described by the heatmap in Figure 4C, revealed that for basal BC the two miRNAs were significantly correlated with some IFs. *miR-135b* was correlated with Kurtosis f intensity histogram (*p*-value < 0.05), and *miR-365-2* was correlated with long run emphasis (LRE), SRE, and LRLGLE (*p*-value < 0.05) from GLRLM. For this subtype, further investigations are required due to the limited number of basal samples.

In conclusion, we found 15 statistically significant miRNAs associated with IFs. In Luminal A samples, we found six miRNAs (*miR-1537*, *miR-205*, *miR-335*, *miR-337*, *miR-452*, and *miR-99a*) correlated with both morphological features and features extracted using intensity histogram analysis and texture analysis. Despite further investigations required in other subtypes, limited by the reduced sample size, seven miRNAs (*miR-142*, *miR-155*, *miR-190*, *miR-190b*, *miR-1910*, *miR-3617*, and *miR-429*) in the HER2+ subtype and two miRNAs in the basal subtype (*miR-135b* and *miR-365-2*) were found to be correlated with types of Ifs.

### 2.2. Diagnostic Role of RadiomiRNomic Signature

To identify a radiomiRNomic signature able to classify Luminal A BC vs. all other subtypes, we analyzed the classification performance of single miRNAs (Table 1), IFs (Table 2), and a combination of multiple miRNA and IFs (Table 3). For this purpose, we used the TCGA/TCIA-BRCA_1 dataset, containing both IFs and miRNA expression levels.

Of the 15 signatures of single miRNAs, 10 performed the best in the TCGA/TCIA-BRCA_1 dataset, with an area under the curve (AUC) > 0.60 (Table 1, column 1). *miR-190b*, found to be a miRNA regulating pathway cross-talks in HER2+ BC (axonal guidance signaling pathway, CXCR4 signaling, and P2Y purigenic receptor signaling pathway) obtained the best AUC performance of 0.92. *miR-155*, the miRNA regulating pathway cross-talk in HER2+ BC (growth hormone signaling and role of macrophages, fibroblasts, and endothelial cells in R.A.), achieved an AUC value of 0.88. *miR-337,* the miRNA regulating pathway cross-talk in luminal A BC (acute phase response signaling and axonal guidance signaling pathways) achieved an AUC value of 0.87. *miR-135-b*, the miRNA regulating pathway cross-talk in basal BC (mismatch repair signaling and ethanol degradation IV pathways) achieved an AUC of 0.73. These signatures of single miRNAs were validated, from imaging, on the GSE81000 dataset. Three miRNAs obtained an AUC value around or above 0.70 (Table 1, column 2). These miRNAs were used for the validation on human BC samples (see below).

Of the 10 signatures of single IFs, five performed the best in the TCGA/TCIA-BRCA_1 dataset, with an AUC > 0.60 (Table 2, column 1). Overall, in the TCGA/TCIA-BRCA_1 dataset, miRNAs seem to provide better classification than IFs, but this is probably related to the sample size used for epigenetic analysis with respect to the sample size of the image’s dataset. Three IFs (correlation from GLCM, SRE, and volume of enhancement) achieved an AUC above 0.60 in the independent dataset TCGA/TCIA-BRCA_2 (Table 2, column 2) 

We expected to obtain much lower AUC values on the test sets than on the training sets. We obtained some AUCs with higher values due to the low number of samples in the training set. However, subsequent studies on datasets containing more samples must be performed. The best performance was obtained when miRNAs and IFs are matched into a combined signature (Table 3 and Table 4); to validate the obtained results, an independent and combined dataset is required, which is not yet available.

### 2.3. Validation of the Three miRNA Signature for the Differential Diagnosis of Human BC Samples

**Figure 5:**
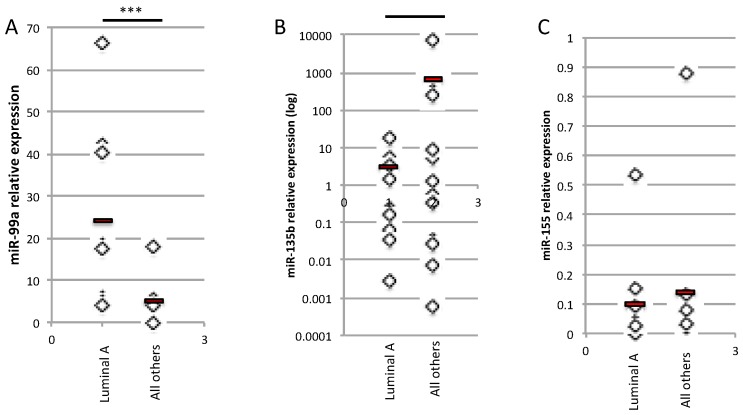
Relative expression of *miR-99a* (**A**), *miR-135b* (**B**) and *miR-155* (**C**) in Luminal A vs. all the other BC subtypes (Luminal B, Basal, HER2+) (*n* = 9) (*** *t*-test, *p*-value < 0.001).

As *miR-99a*, *miR-135b,* and *miR-155* were the miRNAs of the radiomiRNomic signature that produced the best classification performance in terms of AUC on the GSE81000 validation dataset and, in combination with IFs, performed good classification on the AUC TCGA/TCIA-BRCA_1 (Table 4), we performed real-time (RT)-PCR analysis on human BC samples as a validation test. We compared the relative expression level of each miRNA in Luminal A BC vs. all the other BC subtypes, including Luminal B, HER2+, and basal BC tissues (*n* = 9). As shown in Figure 5, the *miR-99a* expression level is reduced in Luminal B, HER2+, and basal BC tissues compared with Luminal A BC, as expected (Figure 5A). As predicted from the bioinformatics analysis, the expression levels of *miR-135b* and *miR-155* were upregulated in Luminal B, HER2+, and basal BC tissues compared with Luminal A BC (Figure 5B,C, respectively).

## 3. Discussion

To identify a radiomiRNomic signature able to classify Luminal A vs. the other BC subtypes, we comprehensively analyzed the integration of miRNomic and radiomic data of BC subtypes from TCGA and TCIA. To the best of our knowledge, this is the first report combining multiple types of miRNomic data integrated with pathway networks and radiomic data in BC, assessing the associations between miRNomic and radiomic features and four BC subtypes related to different prognoses, and evaluating miRNAs and imaging signatures to stratify patients with the Luminal A histological type. A few studies associated image phenotype of intratumor heterogeneity to omics and clinical data [11,23]. The limitation of these analyses is the low number of patients [15].

We demonstrated that an association exists between IFs and miRNAs controlling several potential targets. We identified the functional pathways containing a higher number of miRNA-regulated DEGs, correlated with several parameters of IFs, highlighting the direct link between the specific miRNomic phenotype and the phenotype described by imaging of BC lesions. Different miRNAs were found to be correlated with parameters describing the heterogeneity of the vascularization pattern of the tumor.

In Luminal A, we found some correlations between parameters describing the heterogeneity of the vascularization pattern of the tumor and *miR-99a*, *-205*, *-335*, *-337*, and *-452*, suggesting that these miRNAs could play a main role in the control of mRNA-encoding proteins involved in this process. In particular, we identified acute phase response signaling and axonal guidance signaling pathways that describe the immunological acute phase response activation [31,32] and the hyperproliferation and invasion [33,34] of this BC subtype, respectively. Further investigations are required to determine how these imaging parameters could be considered surrogate features of the immunological response or of the cell proliferation. Moreover, the accuracy of this parameter should be validated to stratify Luminal A patients in a more extended dataset, since only limited accuracy was obtained with our reduced validation set. From a biological point of view, it is important to investigate how the indicated miRNAs could be involved in the control of heterogeneity of tumor vascularization.

In Luminal B, HER2+ and basal subtypes, we found some correlations between miRNomic and imaging phenotypes. Although the associated miRNAs may be involved in tumor vascularization, the limited number of samples in the analysis prevented straightforward interpretation of the obtained results.

Despite the limited number of patients, different IFs were correlated with miRNAs in the different BC subtypes. This suggests that biological pathways, which may be important in each of the different subtypes, can drive the choice of the optimized imaging modality reflecting the characteristics of each different subtype.

Considering the association between the imaging parameters and the miRNAs, we applied the obtained imaging and miRNA-correlation results to the differential diagnosis of Luminal A compared with the other BC subtypes to determine the radiomiRNomic combination able to classify non metastatic BC (Luminal A) versus potentially metastatic BC (all other subtypes). In particular, miRNAs and IFs that were found to be associated with the different BC subtypes were used to evaluate the classification performance of several radiomiRNomic signatures for the differential diagnosis of Luminal A BC. We analyzed the classification performance of single miRNAs or IFs and we evaluated the performance ability of the combination of multiple miRNA and IFs. As a first result, we observed that single miRNAs were more predictive in the classification of Luminal A BC than single radiomic features. This result has to be confirmed, since it could be an effect of disproportion in validation between the sample sizes of the images dataset used for validation (15 Luminal A BC vs. 12 other subtypes in TCGA/TCIA-BRCA_2) with respect to the sample size of epigenetic data (155 Luminal A BC vs. 176 other subtypes in GSE81000).

When combining miRNAs and IFs, better performance was achieved with an AUC value of 0.94. This result has to be confirmed with a validation test in an independent and combined dataset, but it suggests that a combination of miRNAs and imaging parameters as baseline can be used for patient stratification before surgery or chemotherapy treatment.

Considering the possibility of evaluating miRNAs in serum, our results suggest that the methodological approach proposed in this work has the potential to impact the clinical workup of BC patients, enhancing diagnostic confidence using minimally invasive procedures, such as MRI and blood sampling.

An extension to this work could be testing and comparing our radiomiRNomic signature with other classification algorithms such as random forest classification k-nearest neighbor (NN) or an improved version of k-NN [35].

## 4. Materials and Methods

### 4.1. Genomic and Imaging Datasets

In this study, we focused on the gene and miRNA expression levels of four different BC subtypes: Luminal A, Luminal B, HER2+, and basal. We used the following datasets to derive radiomiRNomic signature:

(1) TCGA-BRCA: Gene and miRNA expression levels derived by The Cancer Genome Atlas (TCGA) were downloaded including the profiles of 233 BC Luminal A, 103 BC Luminal B, 43 BC HER2+, and 74 BC basal patients, according to PAM50 classification. BC subtypes were compared with 113 normal samples, allowing the identification of the DEGs of each subtype.
(2) TCGA/TCIA-BRCA_1 dataset: We used miRNA expression profiles of the TCGA-BRCA’s subset to explore the relationships between miRNAs and radiological phenotype. The subset of TCGA-BRCA (TCGA/TCIA-BRCA) was selected to include patients acquired on the same MR scanner to avoid the impact of scanner on imaging features and it contains the genomic profiles and images of 24 Luminal A, 4 Luminal B, 3 HER2+, and 6 basal samples. For these MRI samples, miRNA expression levels and PAM50 classification were available. This analysis allowed the identification of radiomic features of each BC subtype.

**Figure 6:**
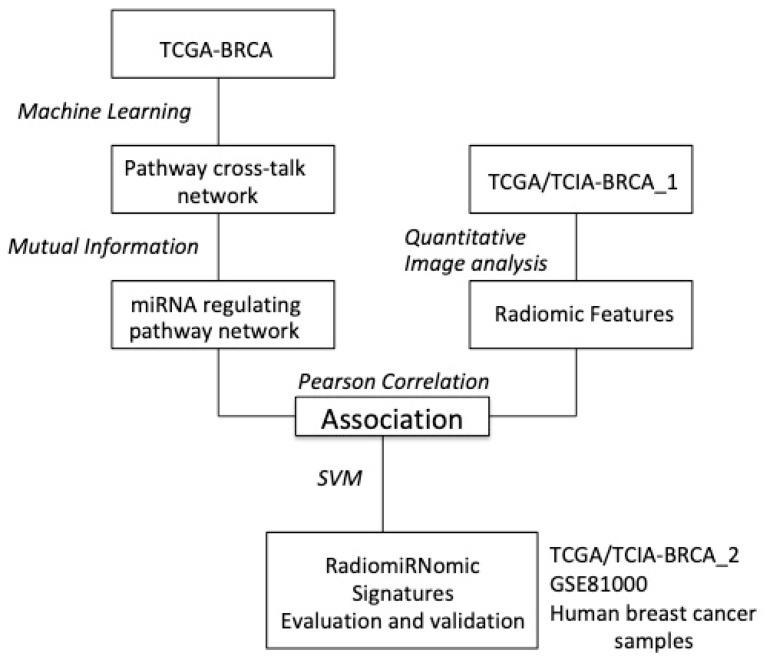
Workflow of the proposed approach.

Since an independent dataset containing miRNA and MRI images was not available, we used the following datasets to independently validate the obtained results: To validate the radiomic signature, we used the TCGA/TCIA-BRCA_2 dataset. This independent subset, which contains 15 Luminal A, 5 Luminal B, 3 HER2+, and 4 basal samples, was not used in the previous analyses. To validate the miRNomic signature, we used the independent GSE81000 dataset, which consists of 155 Luminal A, 89 Luminal B, 42 HER2+, and 45 basal samples. We used this data set to validate the diagnostic value of the miRNomic features in a second cohort of BC patients. A scheme of the followed approach is depicted in Figure 6.

The radiomic and miRNomic signatures were combined and tested on the TCGA/TCIA-BRCA_1 dataset, allowing the identification of radiomiRNomic signatures able to best classify Luminal A versus all other BC subtypes (including Luminal B, HER2+, and basal BCs). TCGA data were normalized using the TCGAbiolinks package [36].

### 4.2. TCGA/TCIA-BRCA_1 Dataset: Imaging Data Analysis

DCE-MRI data were downloaded and analyzed from TCIA, selecting from the multicentric database only images acquired on a Signa Excite GE scanner (Oxford Instruments Healthcare, Ann Arbor, MI) with 1.5 T magnet strength were considered. DCE-MRI images were acquired using gadolinium-based contrast agent, and one pre-contrast and three to five post-contrast images were obtained in the imaging protocol. Subtraction images were generated between the first post-contrast images and the pre-contrast acquisition.

Breast masses were segmented on subtraction images using an approach based on region growing implemented in 3D Slicer. IFs were extracted from subtraction images using the HeterogeneityCAD package in open source 3D Slicer. The analysis included the extraction of 16 radiomic features from image histogram analysis (IF_h_), 9 radiomic features from analysis of tumor morphology and shape (IF_m_), and 32 textural features from the gray-level co-occurrence matrix (IF_GLCM_) and gray-level run length matrix (IF_GLRLM_). A total of 57 imaging features were extracted from subtraction images and, as a preliminary step, feature selection was conducted to avoid feature redundancy. Non-redundant features were selected for correlation analysis by calculating the Spearman correlation coefficients of pairs of features, both within each group and with different groups of features. Clusters of highly correlated features were collapsed into one representative feature on the basis of their inter-subject variability, as expressed by the feature coefficient of variation [15].

A set of 16 features were selected and used to study the association with miRNAs (Table 5).

### 4.3. TCGA-BRCA Dataset: Analysis of miRNAs Regulating Pathway Cross-Talk in BC Subtypes

We applied an algorithm and approach previously presented [37,38] to the TCGA-BRCA dataset to identify miRNAs regulating pathway cross-talk for each BC subtype. Given 589 pathways derived using ingenuity pathways analysis, we filtered the pathways to select the differentially expressed pathways between BC subtypes and normal samples. We quantified the cross-talk between these pathways with a discriminating score. This score was used to select the pathway cross-talk network that achieves the best performance in the classification of BC samples vs. normal samples using a machine learning approach. Since we obtained the networks of pathways for each BC subtype, we focused on the role of miRNAs in regulating miRNA-R networks.

### 4.4. TCGA/TCIA-BRCA_1 Dataset: Association Between miRNAs and IFs

In this step, we revealed the association of IFs with miRNA-R. For each IF, we calculated the Pearson correlation with miRNA-R expression levels. Considering the corresponding *p*-values of the correlation, only IFs and miRNAs significantly correlated (*p*-values < 0.05) were retained for subsequent analysis.

### 4.5. TCGA/TCIA-BRCA_1, TCGA/TCIA-BRCA_2, and GSE81000 Datasets: Diagnostic Role of RadiomiRNomic Signature

Significantly correlated IFs and miRNAs were combined and tested on the TCGA/TCIA-BRCA_1 dataset to define radiomiRNomic signatures for differential diagnosis of Luminal A BC. Given the higher probability of developing a metastatic status in Luminal B, HER2+, and basal samples, we grouped the data in two sets: one set containing 24 BC samples (Luminal A) and a second set containing 13 samples (Luminal B, HER2+, and basal). The performance was evaluated using area under the receiver operating characteristic (ROC) curve (AUC). We evaluated the performance of single miRNAs, single IFs, and combination of miRNAs and IFs using AUC values. AUC values were indicated only for those datasets where miRNA or imaging features were present.

Since an independent dataset of combined miRNAs and MRI images was not available for validation, we performed the validation of the obtained results independently. Validation for IFs was performed on the TCGA/TCIA-BRCA_2 dataset considering 15 Luminal A patients and 12 samples of the other BC subtypes. Validation of miRNAs was performed on the GEO dataset (GSE81000), using 155 BC samples of Luminal A patients and a second set containing 176 samples of the other BC subtypes.

For the evaluation and validation of signature, we developed and used a support vector machine (SVM) using the R-package e1071. We optimized the SVM-feasible learning parameters: cost = 10^(–1:2)^, γ = c (0.5,1,2); kernel type = RADIAL (see e1071 documentation [20]) [39] to identify the best parameters for the SVM learning process. These parameters were used to classify the testing set.

We implemented a Monte Carlo cross-validation method, which randomly selects some fractions of data (60% of the original dataset) to form the training set, and then assigns the rest of the samples to the testing set (40% of the original dataset).

### 4.6. Validation on BC Human Tissue Samples

As we did not have an independent dataset containing IFs and miRNAs for the validation phase, we decided to validate miRNA-R expression levels directly on 9 Luminal A tissue samples compared to 9 tissue samples of other subtypes (including Luminal B, HER2+, and basal human tumor tissues) from surgical resections performed from 2011 to 2013 at the Breast Unit of Istituti Clinici Scientifici Maugeri IRCCS, Pavia, Italy. Samples belonging to the biological collection of the Bruno Boerci Oncological Biobank for research applications (Istituti Clinici Scientifici Maugeri IRCCS, Pavia, Italy), an ISO 9001:2015-certified biobank and member of the Italian node of the Biobanking and BioMolecular Resources Research Infrastructure–European Research Infrastructure Consortium (BBMRI-ERIC; BBMRI.it). Upon receiving patients’ informed consent, samples were collected immediately after surgery, processed, and stored at −80 °C as snap-frozen aliquots according to the best practices in biobanking (certification ISO 9001:2015). At the time of collection, immunohistochemical (IHC) molecular characterization was performed for each subtype by the Pathology Service (Istituti Clinici Scientifici Maugeri IRCCS, Pavia, Italy), according to the clinical guidelines on BC (American Society of Clinical Oncology, ASCO). Molecular characterization has already been published [40]. The samples were used for the isolation of total RNA and for the validation of miRNAs in real-time PCR.

Total RNA was isolated using TRIzol reagent (Life Technologies, Waltham, MA, USA) following the manufacturer’s recommendations and a previous report [9]. For miRNA quantification, total RNA was reverse transcribed using the MystiCq microRNA cDNA synthesis kit (Sigma Aldrich, Merck, Italy), following the manufacturer’s recommendations. miRNAs were amplified in real-time-PCR (RT-PCR) (Eco-Illumina, Euroclone, Italy) using Power Up Sybr green mix (Applied Biosystems, Life Technologies Monza, Italy) in combination with homemade designed primers: *Hsa-miR-99a (miR-99a)* Fwd primer 5′-AACCCGTAGATCCGATCTTGTG-3′, *Hsa-miR-135b-5p (miR-135b)* Fwd primer 5′-TATGGCTTTTCATTCCTATGTGA-3′, and *Hsa-miR-155-5p (miR-155)* Fwd primer 5′-TTAATGCTAATCGTGATAGGGGTT-3′. For each RT-PCR analysis, the results are presented as 2^−^^ΔCt^ method [41], comparing the results of the expression of a housekeeping miRNA, either *miR-103-3p* (5′-AGCAGCATTGTACAGGGCTATGA-3′) or the positive control present in the MystiCq microRNA cDNA synthesis kit (Sigma Aldrich, Merck, Italy). All RT-PCRs were performed in triplicate and the results are the average ± SD of three independent experiments.

## Figures and Tables

**Table 1:** Results of the classification of miRNAs in TCGA/TCIA-BRCA_1 and GSE81000 datasets.

miRNA	AUC-TCGA/TCIA-BRCA_1	AUC-GSE81000
***miR-190b***	0.92	0.56
***miR-155***	0.88	0.74
***miR-337***	0.87	0.65
***miR-135b***	0.73	0.69
***miR-99a***	0.72	0.8
***miR-365-2***	0.68	0.55
***miR-335***	0.66	0.45
***miR-452***	0.64	0.51
***miR-429***	0.62	0.54
***miR-190***	0.61	-

**Table 2:** Results of the classification of imaging features in TCGA/TCIA-BRCA_1 and TCGA/TCIA-BRCA_1 datasets.

Imaging Features	AUC-TCGA/TCIA-BRCA_1	AUC-TCGA/TCIA-BRCA_2
**Correlation (Corr.)**	0.84	0.63
**Short Run Emphasis (SRE)**	0.76	0.66
Long Run High Gray Level Emphasis (**LRHGLE**)	0.7	0.47
**Volume (V)**	0.6	0.78
Sum Average (**SumA**)	0.6	0.5

**Table 3:** Results of the classification of pairwise combination miRNA/imaging features in TCGA/TCIA-BRCA.

miRNA	AUC-TCGA/TCIA-BRCA_1
***miR-135b*; LRHGLE**	0.94
***miR-135b*; V**	0.92
***miR-135b;* SumA**	0.88
***miR-155*; V**	0.86
***miR-135b*; Corr**	0.84
***miR-99a*; SRE**	0.82
***miR-155*; Corr**	0.8
***miR-135b*; SRE**	0.8
***miR-99a*; SumA**	0.74
***miR-155*; SumA**	0.7
***miR-155*; LRHGLE**	0.7

**Table 4:** Results of the classification using combinations of three features (miRNA/imaging features) in TCGA/TCIA-BRCA_1 and miRNAs in GSE81000.

miRNA	AUC-TCGA/TCIA-BRCA_1	AUC-(GSE81000)
***miR-135b; miR-99a*; SRE**	0.94	
***miR-135b; miR-155; miR-99a***	0.94	0.85
***miR-155; miR-99a;* SRE**	0.92	
***miR-135b; miR-155;* SRE**	0.9	
***miR-135b; miR-99a*; Corr**	0.88	

**Table 5:** Sixteen selected features from imaging analysis to be correlated with miRNAs.

Type of Feature	Feature
Histogram-based features	Energy [(g/cc)^2^] – [(mm^2^/sec)^2^]
	Skewness
	Kurtosis
	Median [g/cc] **–** [mm^2^/sec]
Shape-and-size based features	Volume [cm^3^] (V)
	Surface to volume ratio [cm^−1^] (SV)
Gray-Level Co-Occurrence Matrix based features (IF_GLCM_)	Correlation (Corr.)
	Variance (VAR)
	Cluster prominence (CP)
	Cluster tendency (CT)
	Sum Average (SumA)
Gray-Level Run-Length Matrix based features (IF_GLRLM_)	Long Run Emphasis (LRE)
	Short Run Emphasis (SRE)
	Run Percentage (RP)
	Long Run Low Gray Level Emphasis (LRLGLE)
	Long Run High Gray Level Emphasis (LRHGLE)
